# Exploring Virulence Determinants of Filamentous Fungal Pathogens through Interactions with Soil Amoebae

**DOI:** 10.3389/fcimb.2017.00497

**Published:** 2017-12-05

**Authors:** Silvia Novohradská, Iuliia Ferling, Falk Hillmann

**Affiliations:** ^1^Evolution of Microbial Interactions, Leibniz Institute for Natural Product Research and Infection Biology-Hans Knöll Institute, Jena, Germany; ^2^Institute of Microbiology, Friedrich Schiller University Jena, Jena, Germany

**Keywords:** dictyostelium, acanthamoeba, aspergillus, phagocytosis, macrophages, amoebae

## Abstract

Infections with filamentous fungi are common to all animals, but attention is rising especially due to the increasing incidence and high mortality rates observed in immunocompromised human individuals. Here, *Aspergillus fumigatus* and other members of its genus are the leading causative agents. Attributes like their saprophytic life-style in various ecological niches coupled with nutritional flexibility and a broad host range have fostered the hypothesis that environmental predators could have been the actual target for some of their virulence determinants. In this mini review, we have merged the recent findings focused on the potential dual-use of fungal defense strategies against innate immune cells and soil amoebae as natural phagocytes. Well-established virulence attributes like the melanized surface of fungal conidia or their capacity to produce toxic secondary metabolites have also been found to be protective against the model amoeba *Dictyostelium discoideum*. Some of the recent advances during interaction studies with human cells have further promoted the adaptation of other amoeba infection models, including the wide-spread generalist *Acanthamoeba castellanii*, or less prominent representatives like *Vermamoeba vermiformis*. We further highlight prospects and limits of these natural phagocyte models with regard to the infection biology of filamentous fungi and in comparison to the phagocytes of the innate immune system.

## Environmentally acquired fungal pathogens

Fungi are ubiquitous in nature, inhabiting various ecological niches. Even among those which thrive as saprophytes and do not exhibit any host requirement for survival, there are pathogens which cause devastating diseases in humans and animals resulting in thousands of deaths every year (Brown et al., 2012). Classical examples include filamentous fungi like *Aspergillus fumigatus* and *Fusarium* sp., but also several dimorphic fungi such as *Blastomyces dermatitidis* or *Histoplasma capsulatum*, and the yeast *Cryptococcus neoformans*, have environmental reservoirs. One of the most prevalent groups of fungi in the environment is represented by the aspergilli (Shelton et al., 2002). With several hundred species, only a few of them have a considerable impact on human health: *A. fumigatus, A. flavus, A. terreus, A. nidulans*, and *A. niger*.

*Aspergillus fumigatus* is one of the most important air-borne fungal pathogens, living ubiquitously in terrestrial environments. This fungus disseminates by releasing thousands of asexual spores (conidia) from each conidiophore which, upon inhalation, pass through the nasal cavity and reach the alveoli. Most of them are expelled by mucocilliary clearance while the residual ones are eliminated by macrophages and neutrophils of an immunocompetent host. In the case of an immunodeficient host, conidia swell and grow into a mycelium. Once the fungus overcomes the natural immune barrier, it can cause asthma-associated allergies, sinusitis, allergic bronchopulmonary aspergillosis (ABPA) and, in the worst case, life-threatening invasive aspergillosis (IA), occasionally reaching mortality rates even beyond 50% due to rapid progression and misdiagnosis (Brown et al., 2012). Aspergilli can also infect wild and domestic animals nearly encompassing all major phyla including corals, honey bees, reptiles, and warm-blooded animals such as birds, mammals, and non-human primates (reviewed by Seyedmousavi et al., 2015). Another group of environmental filamentous fungi represent exclusively entomogenous pathogens, such as *Metarhizium anisopliae* or *Beauveria bassiana*. Microscopic examination and phagocytosis assays with these fungi suggested that their ability to adhere to the insect cuticle, penetrate through the haemocoel using hydrolyzing enzymes, and ultimately survive phagocytic haemocytes may be a consequence of adaptations that have been acquired early in evolution to avoid predation by soil amoebae (Bidochka et al., 2010).

Unlike *Aspergillus, C. neoformans* is not ubiquitous in the soil; rather it has been isolated from areas frequented by pigeons, chickens, turkeys, and other avian species. After inhalation of infectious particles, *Cryptococcus* resides in the lung alveoli where it can persist and replicate while a thick polysaccharide capsule surrounding the yeast cell helps to avoid its killing by macrophages. Other studies have shown that *Cryptococcus* is also able to survive intracellularly, even a few hour after phagocytosis (Feldmesser et al., 2000). Dissemination to the brain results in severe meningoencephalitis, especially in immunocompromised patients. On the other hand, *C. gatii* which has been isolated from trees, mainly causes pulmonary infections in an immunocompetent host (García-Rodas and Zaragoza, 2012; Kwon-Chung et al., 2014).

Among other environmentally acquired pathogenic fungi, thermally dimorphic fungi such as *Histoplasma capsulatum, Blastomyces dermatitidis*, and *Coccidioides immitis* are especially clinically relevant and classified as Biosafety Level 3 (BSL3) organisms. Despite their divergent phylogeny, they all share similar patterns of existence: temperature-dependent morphological dimorphism, pulmonary infectivity, and endemism. After the inhalation of conidia, transformation into a yeast-form is crucial to promote pathogenicity through escape from phagocytosis, modulation of the cytotoxic environment of the phagolysosome or enhanced degradation of reactive oxygen species (Boyce and Andrianopoulos, 2015).

Considering the diversity of environmental niches and strategies to survive and replicate within a variety of mammalian hosts, the aforementioned virulence attributes may confer a dual-use capability to defend against phagocytes in both animal hosts and the environment. Moreover, such parallels gave rise to the idea that selective pressures in the environment have led to the emergence and maintenance of these traits that have later supported virulence in higher eukaryotes. Although the filamentous life style of aspergilli suggests little need for any specific attributes to avoid or withstand any phagocytes, their infectious and reproductive stage is formed by small, unicellular conidia which are easily ingested by such cells. A number of profound studies over the last years have uncovered a multitude of mechanisms which aid in the escape and defense of these fungi against their opponents of human or environmental origin (Table 1).

**Table 1:** Fungal virulence determinants studied using amoeba model systems.

**Amoeba model**	**Fungal pathogen**	**Virulence factor studied**	**References**
**HUMAN PATHOGENIC FUNGI**			
*A. castellanii*	*C. neoformans*	Capsule, melanin, phospholipase production	Steenbergen et al., 2001; Chrisman et al., 2010
		Comparative transcriptomic study	Derengowski et al., 2013
		Extracellular vesicles, glucuronoxylomannan of capsule	Rizzo et al., 2017
	*H. capsulatum*	Yeast-to-hyphae transition	Steenbergen et al., 2004
	*S. schenckii*		
	*B. dermatitidis*		
	*A. fumigatus*	Phagocytic escape	Van Waeyenberghe et al., 2013
*A. castellanii* *Naegleria gruberi*	*A. fumigatus* *A. terreus*	Diffusible compound with anti-amoebic properties	Hobson, 2000
*D. discoideum*	*C. neoformans*	Capsule, melanin	Steenbergen et al., 2003
	*A. fumigatus*	DHN-melanin, gliotoxin	Hillmann et al., 2015
		Trypacidin	Mattern et al., 2015
	*A. terreus*	Asp-melanin	Geib et al., 2016
	*S. cerevisiae* *C. albicans* *C. glabrata*	Flocculation Hyphae formation	Koller et al., 2016
**ENTOMOPATHOGENIC FUNGI**			
*A. castellanii*	*M. anisopliae* *B. bassiana*	Phagocytic escape and survival	Bidochka et al., 2010
**OTHER**			
Giant vampyrellid soil amoebae	Various soil-borne species, Plant pathogenic fungi	First feeding trials to assess the ability of soil amoeba to attack, perforate and lyse the spores of different soil fungi	Old and Darbyshire, 1978; Chakraborty et al., 1983; Old et al., 1985; Chakraborty and Old, 1986
*Protostelium mycophaga*			Olive and Stoianovitch, 1960

## Fungal resistance to innate immune cells and soil amoebae

Macrophages and neutrophil granulocytes are the most prominent representatives among innate immune cells which counteract fungal pathogens. Alveolar macrophages are the first line of defense against IA by killing inhaled conidia and initiating the pro-inflammatory response that recruits neutrophils to the site of infection (reviewed by Brakhage et al., 2010). As a consequence, patients with reduced numbers of macrophages have long been known to be at a higher risk to develop IA (Brakhage, 2005). A number of *in-vitro* studies suggest that even macrophages from immunocompetent individuals show variable killing efficiencies of ingested fungal conidia ranging from 10 to 90% as summarized in Philippe et al. (2003). The same study demonstrated that fungal conidia are especially resistant to killing when remaining in a dormant state or when the confronting macrophages are derived from immunosuppressed donors. Overall, most of these results are based on counts of colony forming units (CFUs) and certain methodological issues, such as the time of co-incubation, conidial aggregation or the incomplete experimental removal of non-ingested conidia, could have also contributed to the heterogeneous killing rates that have been reported. A similar assay with *Dictyostelium discoideum* revealed that conidia were readily ingested but remained viable over more than 24 h based on the number of CFUs (Hillmann et al., 2015). Even the comparably robust pathogen *Acanthamoeba castellanii* ingested conidia within the first hour of their interaction, but no signs of digestion were observed at any stage (Van Waeyenberghe et al., 2013). Along this line, co-incubation of *A. fumigatus* or *Fusarium oxysporum* conidia with the common water contaminant *Vermamoeba vermiformis* did not result in any reduction of viable conidia, but instead phagocytic uptake promoted filamentation and growth of the fungus (Cateau et al., 2014; Maisonneuve et al., 2016).

## Recognition and processing of fungal conidia by human and environmental phagocytes

**Figure 1:**
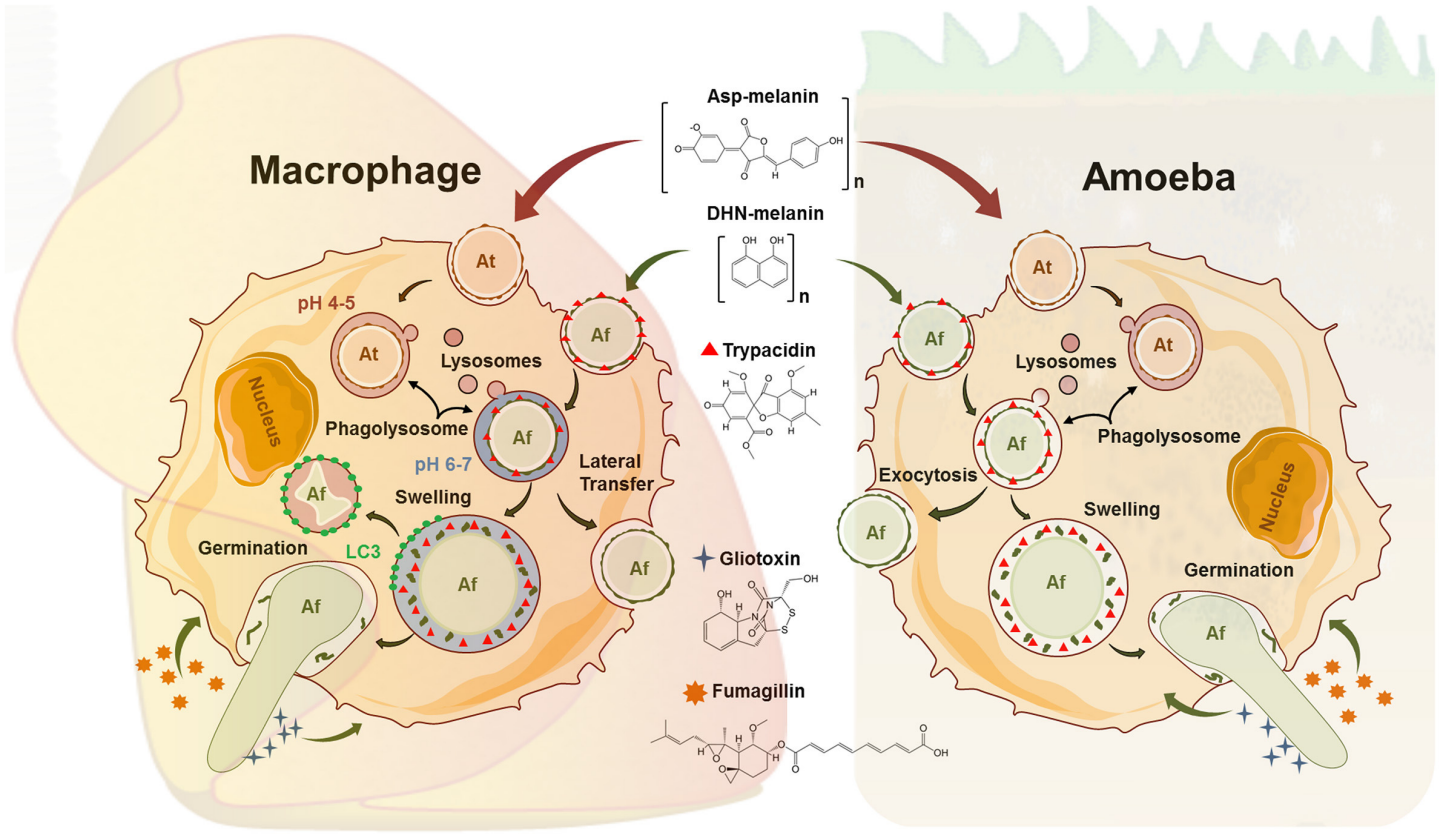
Comparative schematic view on parallel events in the phagocytic processing of fungal conidia from *Aspergillus fumigatus* (Af) and *Aspergillus terreus* (At) in macrophages and amoeba. The latter summarizes results from *A. castellanii* (Ac) and *D. discoideum* (Dd). The conidial pigments Asp-melanin (At) and DHN-melanin (Af) are complex polymers and the final-known intermediate structure is displayed. Ingested spores of At can persist in acidified phagolysosomes (PLs) in macrophages. Acidification of At containing PLs occurs also in *D. discoideum*. In macrophages, Af can either be killed *via* the LC3 dependent pathway, laterally transferred to other cells, or undergo swelling and germination. Exocytosis (Ac), swelling (Ac, Dd) and germination (Ac, Dd) of Af conidia has also been documented in amoeba, while killing by amoeba has not been reported. The spore-borne trypacidin, and the secreted gliotoxin and fumagillin are all made by *A. fumigatus* only and are all known to affect macrophages as well as Dd.

Given their role as a first line of defense, studies on the recognition and phagocytic processing of fungal conidia by macrophages have a long-standing history, mainly with regard to aspergilli. The surface of their conidia represents the immediate interface and pathogen-associated molecular patterns (PAMPs) include cell wall constituents like α- and β-glucans, chitins, galactomannans, and other polysaccharides. However, these are usually masked by a proteinaceous, hydrophobic rodlet layer which is immunologically inert and diminishes the recognition by immune cells (Aimanianda et al., 2009). The green-gray dihydroxynapthalene (DHN)-melanin pigment coating dormant conidia is another surface component assumed to play a similar role in *A. fumigatus* (Jahn et al., 1997; Tsai et al., 1998; Chai et al., 2010). These protective layers are lost during swelling and subsequent germination of the conidia (Figure 1), exposing the PAMPs and allowing recognition, as demonstrated for strains of *A. fumigatus* lacking the DHN melanin pigment which were ingested by macrophages at higher rates than wild type strains (Luther et al., 2007). Interestingly, nearly identical ratios were observed with *D. discoideum*. Conidia of the wild-type, covered by green DHN-melanin, were taken up, but at least threefold less efficiently than the white conidia of the melanin deficient mutant (Hillmann et al., 2015). These data suggest that hiding “prey”-associated molecular patterns could be an asset to escape also from environmental predators and hence, well suited to be studied in an amoeba model.

Swollen conidia of *A. fumigatus* that have lost their DHN-melanin cover expose cell wall β-glucans which activate the Dectin-1/Syk kinase/NADPH signaling cascade in macrophages (Luther et al., 2007; Ma et al., 2012). After recognition and internalization, phagolysosome maturation is initiated through assembly of the NADPH oxidase and LC3-associated phagocytosis (LAP) (Akoumianaki et al., 2016). Melanized conidia of *A. fumigatus* are able to inhibit the crucial process of phagolysosomal maturation at the acidification step which contributes to their significantly reduced killing rates relative to melanin deficient conidia (Jahn et al., 2002; Thywißen et al., 2011, Figure 1). In sharp contrast, melanized conidia were shown to inhibit the apoptosis of macrophages by activating the PI3-kinase/Akt signaling pathway (Volling et al., 2011).

The chemically distinct Asp-melanin of *Aspergillus terreus* does not inhibit phagolysosomal acidification in macrophages or *D. discoideum* (Figure 1), indicating that even closely related fungi, such as *A. fumigatus* and *A. terreus*, apply different persistence and propagation strategies inside the harsh phagolysosomes of macrophages and some environmental phagocytes (Slesiona et al., 2012; Geib et al., 2016).

## Phagocyte-escape mechanisms of *A. fumigatus*

Apart from conidial killing, macrophage encounters with *A. fumigatus* may also result in disruption of the host cell by the elongation of the hyphae (Figure 1). Here, *Aspergillus fumigatus* conidia swell up and initiate growth despite the nutrient-scarce environment of the phagolysosome thereby exploiting their glyoxylate cycle and siderophore machinery to overcome the limited supply of carbon and iron, respectively (Behnsen et al., 2007; Schrettl et al., 2010). It seems likely that these pathways could also be active during conidial processing in environmental phagocytes as this escape strategy was well documented during the interaction with *A. castellanii* as well as *D. discoideum*, resulting in host cell lysis for both amoebae (Van Waeyenberghe et al., 2013; Hillmann et al., 2015). Some of the consequences of intracellular fungal germination on the host cell were only recently resolved for human alveolar macrophages. Orchestrated by calcineurin, the infected host cell can complete exocytosis of the fungus containing endosome, followed by the lateral transfer to neighboring cells as part of programmed necrosis (Shah et al., 2016). In perspective of the well-studied social behavior of *D. discoideum*, this amoeba model seems to be especially promising for future studies on such intercellular or altruistic processes. It has become obvious that filamentous fungi can be tough opponents of innate immune cells, but questions on the origin of such anti-immune properties of filamentous fungi remain open. Amoeba models could help to close the knowledge gaps which cannot be addressed from *in vitro* studies with human cells.

## Amoebae predation as an evolutionary training ground for filamentous fungal pathogens

Free living amoebae (FLA) are ubiquitous unicellular protozoa, distributed worldwide in various environments such as soil, water, or air. Mycophagous species, either opportunists or specialists, are widespread and have been isolated over the past 60 years (Olive and Stoianovitch, 1960; Old and Darbyshire, 1978; Chakraborty et al., 1983; Old et al., 1985; Chakraborty and Old, 1986). One of the first mycophagous amoebae ever described was *Protostelium mycophagum* (Olive and Stoianovitch, 1960). It has been isolated alongside the pink-pigmented yeast *Rhodotorula mucilaginosa*, but it can also feed on *Phoma* sp., *Ustilago violacea, Sporobolomyces* sp., and *Cryptococcus laurentii* (Spiegel et al., 2006). Other mycophagous soil amoebae from the genera *Thecamoeba, Arachnula*, and *Vampyrella* have been shown to suppress the growth of the plant pathogenic fungus *Gaeumannomyces graminis* var. *tritici* and therefore significantly contribute to the reduction of “take-all” wheat crops disease caused by this fungus (Chakraborty et al., 1983).

In environments where fungi have encountered constant protozoal predation and competition for nutrients, they must have developed strategies to counteract phagocytic uptake or intracellular passage. Consequently, the same determinants that were effective against amoeba predation, could have later promoted the survival of fungi in a human host. The coincidental evolution hypothesis suggests that virulence factors have evolved as a response to more ordinary selection pressures than for virulence *per se* (Erken et al., 2013). Several studies using free-living amoeba *A. castellanii* and more recently *D. discoideum* have supported this hypothesis for yeast-like fungi and have underlined the suitability of these models to study basic fungal virulence determinants (Steenbergen et al., 2001, 2004; Chrisman et al., 2010; Derengowski et al., 2013; Koller et al., 2016; Rizzo et al., 2017, see Table 1 for an overview).

*Aspergillus fumigatus* requires no obvious residence inside the mammalian host to survive and replicate; it appears to lack classical virulence factors and its pathogenicity primarily depends on host impairment. Therefore, it is conceivable that the ability to counteract the compromised immune system was partially tuned from the long-term interplay between fungi and their predators in their natural environment. Such fungivorous organisms are by no means limited to amoebae, but also include higher animals like nematodes, mites, or insects. For instance, a primary study on *A. nidulans* demonstrated that the multitude of fungal low-molecular-mass compounds known as secondary metabolites could present a selective advantage against predation by the fungivorous springtail *Folsomia candida* (Rohlfs et al., 2007).

Such defensive actions could also provide protection against microscopic predators, as active components have been shown to diffuse from the non-germinating spores and inhibit certain functions of phagocytosis (Slight et al., 1996). The anti-amoebae effects of diffusates from clinical and environmental isolates of *A. fumigatus* and *A. terreus* have been described on *Naegleria gruberi*, proposing it as a primary function of such metabolites (Hobson, 2000). Even at the early stages of a direct interaction, mycotoxins are encountered immediately by the ingesting phagocytes, as it has recently been shown for the amoebicidal polyketide trypacidin which resides primarily on the surface of the *A. fumigatus* spore (Gauthier et al., 2012; Mattern et al., 2015, Figure 1). Following germination and escape, further potent, soluble toxins are synthesized.

Among them, the sesquiterpene fumagillin was one of the first for which amoebacidal properties were observed and has initially been used for the treatment of infections caused by *Entamoeba histolytic*a (Killough et al., 1952). Fumagillin (Figure 1) and its synthetic analogs thereby irreversibly inhibit the methionine aminopeptidase-2 (MetAP2), making them promising therapeutic candidates against malaria parasites, trypanosomes, or other amoebae (Arico-Muendel et al., 2009). When using *D. discoideum* as a model, however, cytotoxic effects on the phagocytes could largely be attributed to the non-ribosomal peptide gliotoxin (Hillmann et al., 2015). The toxic and immunosuppressive properties of gliotoxin, the prototype of the epidithiodioxopiperazine (ETP)-type mycotoxins, are directed toward the host's immune effector cells *via* the activity of its unusual intramolecular disulfide bridge (Figure 1). Several target molecules for gliotoxin have been well described, including the NADPH oxidase of polymorphonuclear leukocytes or central regulatory hubs like the phosphatidylinositol 3,4,5-trisphosphate metabolism and the transcription factor NFκB (Pahl et al., 1996; Tsunawaki et al., 2004; Schlam et al., 2016). Among these studies, Schlam and colleagues have shown that gliotoxin further prevents integrin activation in immortalized and primary macrophages and interferes with actin dynamics. As both of these are essential instruments during phagocytosis and membrane ruffling, such pathways may be attractive targets in the defense against FLA. Previously it was thought that gliotoxin production is restricted only to clinical isolates of *A. fumigatus*; however, it was demonstrated recently that the vast majority (>96%) of both environmental and clinical isolates of aspergilli are able to produce this mycotoxin (Kupfahl et al., 2008; Scharf et al., 2012). Consequently, it is only plausible to suspect that fungi have maintained their whole repertoire of active secondary metabolites to counteract not only their numerous competitors, but also predators in their natural environment whose numbers and diversity have long been underestimated. A recent study supports this conclusion by demonstrating that mycophagous protists are abundant, taxonomically widespread, and central ecological players in the soil food web (Geisen et al., 2016).

## Perspectives

Both *Dictyostelium discoideum* and *Acanthamoeba castellanii* have been extensively studied as model organisms in terms of phagocytic interactions, mainly due to the similarity with human macrophages (Tosetti et al., 2014). As unicellular eukaryotes, with a compartmentalized cytoplasm, relatively small size, and active chemotactic movement they exemplify an ideal non-mammalian model for host-pathogen interactions and microbial infections. Both amoebae are easy to cultivate (either on bacteria or axenically), giving high cell yields with defined identity. Besides, working with these model organisms is highly advantageous in terms of genetic malleability combined with a profound knowledge of its phagocytic pathways (Eichinger et al., 2005; Siddiqui and Khan, 2012). A major advantage of *D. discoideum* over *A. castellanii* is the well annotated genome, the high number of molecular tools and protocols, and the wide availability of targeted mutants that are easily accessible at www.dictybase.org (Eichinger et al., 2005; Bozzaro and Eichinger, 2011; Fey et al., 2013). However, the fact that prolonged subcultivations and axenization of native amoeba cultures may have led to the accumulation of undesired mutations and loss of original phagocytic abilities has to be taken into consideration. Another drawback of *D. discoideum* is its limited maximal survival temperature of roughly 27°C, which may be unfavorable for some fungi that may express their full virulence potential only at temperatures around 37°C. However, at present there is little support for the idea that filamentous fungi like *A. fumigatus* tend to regulate their general virulence attributes strictly in response to higher temperatures. In fact, a recent study revealed that 11 of 37 clusters encoding for the biosynthesis of secondary metabolites were activated by a temperature shift from 37 to 30°C, including those coding for DHN-melanin, gliotoxin, and trypacidin (Lind et al., 2016). Nevertheless, when using amoebae as a tool to study the origin of fungal virulence, it is important to keep in mind that throughout their evolution, fungi have also encountered many other soil-dwelling predators that may have contributed to the emergence of fungal virulence determinants. Certainly not all virulence traits are equally beneficial to protect fungi in different hosts, giving rise to a number of different invertebrate animal models which have been used to study fungal pathogenesis (Desalermos et al., 2012). Very little is known about the interaction of soil amoebae and fungi in their natural environment. It is plausible, that not every amoeba is amenable to infection by every fungal pathogen. Furthermore, bacteria are the preferential prey for *D. discoideum*, while *A. castellanii* was originally discovered as a contaminant in yeast cultures (Castellani, 1930). Even if fungal infection biologists have successfully exploited these two established amoeba models, further studies on the evolution of fungal virulence may call for an inclusion of natural fungivorous predators from the Amoebozoa phylum.

## Author contributions

SN, IF, and FH wrote the initial version of the manuscript. IF and SN prepared illustrations. FH revised the manuscript. All authors read and approved the final version.

### Conflict of interest statement

The authors declare that the research was conducted in the absence of any commercial or financial relationships that could be construed as a potential conflict of interest.
